# Interleukin-17 plays a role in dental pulp inflammation mediated by zoledronic acid: a mechanism unrelated to the Th17 immune response?

**DOI:** 10.1590/1678-7757-2023-0230

**Published:** 2023-10-09

**Authors:** Anna Clara Aragão Matos CARLOS, José Vitor Mota LEMOS, Marcela Maria Fontes BORGES, Maria Carolina Portela ALBUQUERQUE, Fabrício Bitu SOUSA, Ana Paula Negreiros Nunes ALVES, Thinali Sousa DANTAS, Paulo Goberlânio de Barros SILVA

**Affiliations:** 1 Universidade Federal do Ceará Departmento de Patologia Oral Fortaleza Ceará Brasil Universidade Federal do Ceará, Departmento de Patologia Oral, Fortaleza, Ceará, Brasil.; 2 Centro Universitário Christus Departamento de Patologia Fortaleza Ceará Brasil Centro Universitário Christus, Departamento de Patologia, Fortaleza, Ceará, Brasil.

**Keywords:** Zoledronic acid, Dental pulp, Acute-phase reaction, Inflammation

## Abstract

**Objective:**

To evaluate the influence of RORγT inhibition by digoxin on inflammatory changes related to interleukin-17 (IL-17) in the pulp of rats treated with zoledronate (ZOL).

**Methodology:**

Forty male Wistar rats were divided into a negative control group (NCG) treated with saline solution, a positive control group (PCG) treated with ZOL (0.20 mg/kg), and three groups treated with ZOL and co-treated with digoxin 1, 2, or 4 mg/kg (DG1, 2, and 4). After four intravenous administrations of ZOL or saline solution in a 70-day protocol, the right molars were evaluated by histomorphometry (number of blood vessels, blood vessels/µm^2^, cells/µm^2^, total blood vessel area, and average blood vessel area) and immunohistochemistry (IL-17, TNF-α, IL-6, and TGF-β). The Kruskal-Wallis/Dunn test was used for statistical analysis.

**Results:**

PCG showed an increase in total blood vessel area (p=0.008) and average blood vessel area (p=0.014), and digoxin treatment reversed these changes. DG4 showed a reduction in blood vessels/µm^2^ (p<0.001). In PCG odontoblasts, there was an increase in IL-17 (p=0.002) and TNF-α (p=0.002) immunostaining, and in DG4, these changes were reversed. Odontoblasts in the digoxin-treated groups also showed an increase in IL-6 immunostaining (p<0.001) and a reduction in TGF-β immunostaining (p=0.002), and all ZOL-treated groups showed an increase in IL-17 (p=0.011) and TNF-α (p=0.017) in non-odontoblasts cells.

**Conclusion:**

ZOL induces TNF-α- and IL-17-dependent vasodilation and ectasia, and the classical Th17 response activation pathway does not seem to participate in this process.

## Introduction

Bisphosphonates (BPs) are osteolysis inhibitors commonly used in the treatment of degenerative bone diseases such as osteoporosis and Paget’s disease. Recently, the use of this group of drugs in the treatment of bone metastases has gained momentum due to their relatively low cost and their ability to increase the life expectancy and quality of life of patients with malignancies such as multiple myeloma and breast, prostate, kidney, and lung cancers.^1-3^ However, the use of BPs is strongly associated with the development of several immune-inflammatory changes affecting the maxillofacial region.^4^

Zoledronate (ZOL) has a direct toxic effect on many cell groups, including epithelial cells, fibroblasts,^5,6^ osteoblasts,^7^ macrophages,^8,9^ neutrophils,^10^ and endothelial cells.^11^ Cytotoxic changes in these cellular elements are known to activate the production of several pro-inflammatory cytokines. This leaves the ZOL contact site, which is usually close to bone tissue, with a higher inflammatory potential compared with untreated tissues.

Some evidence of ZOL toxicity in the dental pulp is beginning to accumulate. Alendronate can lead to dental teratogenicity^12^ and induces denticle and odontoma formation.^13^ ZOL affects alkaline phosphatase synthesis, changes the cell morphology of odontoblasts,^14^ and also reduces cell viability, proliferation, and protein synthesis in pulp cells in a time-dependent manner.^15^ In a study conducted by our group, it was observed for the first time that animals treated with ZOL showed a dose-dependent pro-inflammatory state in the dental pulp. In this study, even 70 d after the start of ZOL treatment, the dental pulp cells of rats in the experimental group had a high expression of TNF-α, IL-1β, and iNOS. This change was especially prominent in odontoblasts, which are cells in close contact with mineralized dentin material.^16^ In a follow-up study, it was recently observed that other mediators are significantly increased in the dental pulp, such as COX-2, and even bacterial recognition molecules, such as toll-like receptor 4, are overexpressed after long-term treatment with ZOL.^17^

Although these are tissues of different embryonic origin and with different molecular properties, if there is overexpression of IL-17 in BF-treated bone tissue, similar changes are likely to occur in the dental pulp, since IL-17 can sustain the transcription stability of some acute-phase cytokines, such as TNF-α,^1,8^ a cytokine overexpressed in the dental pulp after long periods of ZOL treatment.^16^

Recently, drugs with indications unrelated to the control of inflammation have been described as capable of inhibiting RORγT, leading to a reduction in IL-17 synthesis. Digoxin is a drug that belongs to the glycoside class and is widely used to treat heart failure. Its mechanism of action involves inhibiting the calcium-dependent Na+K+ATPase pump in the cardiac sarcoplasmic reticulum, which raises the intracellular calcium concentration and increases its contraction force.^19^

Due to the ability of digoxin to block RORγT and IL-17 transcription, digoxin may attenuate the overexpression of cytokines in the dental pulp of rats exposed to ZOL. Therefore, this study aimed to evaluate the role of IL-17 inhibition by digoxin in inflammatory events in the dental pulp of rats chronically treated with ZOL.

## Methodology

### Animals, groups, and doses

The Ethics Committee on the Use of Animals (CEUA) of the Christus University Center (UNICHRISTUS) approved this project, registered under number 37/18, and 40 male Wistar rats (*Rattus novergicus*) were used. The rats (180–200 g) were kept with *ad libitum* access to water and food, on a 12-h light-dark cycle, at a temperature of 20–25°C. They were weighed weekly. The rats were randomly divided (“Random” function, Excel 2010, Microsoft Corporation^®^) into five groups: a negative control group (NCG) treated with sterile saline solution (0.1 mL/kg), a positive control group (PCG) treated with ZOL (0.20 mg/kg diluted in 0.1 mL/kg of sterile saline solution), and three test groups treated with ZOL (0.20 mg/kg diluted in 0.1 mL/kg of sterile saline solution) and digoxin 1 (DG1), 2 (DG2), or 4 (DG4) mg/kg diluted in 0.1 mL/kg of sterile saline solution.

### Sample size calculation

A study on an aortic aneurysm model by Wei, et al.^20^ (2014) showed that animals treated with digoxin had reduced levels of IL-17 (0.70±0.07) compared with untreated animals (0.80±0.04). They estimated that it is necessary to have a sample of seven animals per study group to obtain a sample with 90% power and 95% confidence in rejecting the null hypothesis. Considering the possibility of sample loss during the protocol, 10% was added to the calculated sample, totaling eight animals per study group.

### *In vivo* experimental protocol

We used a previously published experimental protocol in which doses infusion simulated administration in humans. Briefly, after using the software Dose Calculator, Conversion Chemotherapy of Humans to Animals, provided free by the Food and Drug Administration (http://www.accessdata.fda.gov), body weight and surface area were the parameters considered for pharmacological conversion of the human dose of ZA for the experimental rats. The monthly dose (4 mg) used to treat multiple myeloma was estimated at 0.60 mg/kg for the Wistar rats and divided into three weekly administrations of 0.20 mg/kg. This dose proved to be a potent inducer of BRONJ in rats. Then, we performed a dose-response curve with little or no toxicity.^21^

The rats underwent three consecutive weekly administrations (days 0, 7, and 14) of ZOL or saline solution (control). On day 49, the fourth dose of the drug was administered and, on day 70, the rats were euthanized. They received sterile saline solution or digoxin at different doses by gavage three times a week from the beginning of the experimental protocol. The first infusion of ZOL was on a Monday (D0), marking the beginning of the experimental protocol. Digoxin administration began on the same Monday and was performed every Monday, Wednesday, and Friday until the end of the experimental protocol proposed by Lee, et al.^22^ (2015), totaling 30 administrations of digoxin.

After euthanasia, the right hemimandibles were surgically excised, stored in 10% neutral formalin for 24 h, then decalcified (suspension) in 10% EDTA solution (pH 7.3; NaOH, PA) for 30 d for histological slides (hematoxylin & eosin, 3 µm).

### Histological analysis of dental pulp: cellularity and vascular events

Histological sections were made using a semi-automatic microtome (Leica^®^) until the pulp chamber was visible in both specimens. Photomicrographs (U-TV0.63XC, Olympus^®^) were taken at 400x magnification (BX43, Olympus^®^ plus Olympus Soft Imagining LCMicro software) and mounted to access the entire dental pulp area of the right mandibular first molars. The images were then mounted and the total pulp area measured in µm^2^ using the Freehand Selection tool in ImageJ^®^ software.

To count the number of cellular elements in the dental pulp, the command Color Deconvolution > Hematoxylin & Eosin was used to segregate the images into three color scales: hematoxylin (nuclei), eosin (cytoplasm and connective tissue), and a residual image. The hematoxylin images were binarized (Image > Binary > Make Binary) to count the number of nuclei (Analyze > Analyze Particles). Cellularity was defined as the number of nuclei/µm^2^.

Using the same Freehand Selection tool, the area of each blood vessel in the dental pulp of the right mandibular first molars of each animal was measured. The sum of these areas was divided by the total area of the dental pulp and expressed as the relative blood vessel area (%).23 The number of vessels in each tooth of each animal was divided by the total area of the dental pulp and expressed as the number of vessels/µm^2^. The average area of each blood vessel was estimated and divided by the total area of the dental pulp of each tooth in each animal, and was expressed as the average blood vessel area/µm^2^.

### Tissue microarray technique (TMA) and immunohistochemistry

To study the immunoexpression of cytokines involved in the Th17 response (IL-17, IL-6, and TGF-β) and their influence on the expression of pro-apoptotic proteins (TNF-α) in the dental pulp of rats, we performed histological and histomorphometric analysis of the dental pulp. A 2 mm diameter microfield from each of these areas was selected and marked on the histological slides for subsequent block pairing and sample removal using a tissue microarrayer device. Then, a recipient block containing 70 wells of the same diameter and depth (2 mm) received paraffin-embedded tissue samples, which were organized and mapped.

After placing all the samples in the recipient block, it was incubated for 3 min on a glass slide in an oven at 65°C and removed with a rotational movement to attach the tissue samples to the recipient block. After cooling the recipient block to room temperature (20–25°C), 3 µm sections were prepared and placed on silanized slides for immunohistochemistry and apoptosis assays.

The silanized slides were deparaffinized in an incubator for 1 h at 65°C, immersed in xylene solution, rehydrated in decreasing alcohol solution, washed in tap water, and subjected to antigenic recovery with 0.1 M citrate solution at pH 6.0 in a water bath at 95°C for 40 min. After cooling to room temperature, the sections were washed in 0.1 M phosphate buffer solution at pH 7.3 (PBS) in two baths for 5 min each, and endogenous peroxidase was blocked with a 3% solution of H_2_O_2_ (hydrogen peroxide) diluted in PBS for 30 min.

After two 5 min PBS baths, the slides were incubated with 1% albumin solution for nonspecific protein blocking for 1 h. After two 5 min washes in PBS, the slides were incubated overnight with the following antibodies: IL-17 (1:100, ab79056; Abcam), TNF-α (1:100, ab6671; Abcam), IL-6 (1:300, ab19324; Abcam), and TGF-β (1:400, ab92486; Abcam).

The next day, after cooling to room temperature, the sections were washed with two 5 min PBS baths and incubated with anti-rabbit/anti-mouse polymer Dako Envision Dual Link System HRP (Dako^®^) for 1 h. After two more 5 min PBS baths, the slides were incubated with 3,3-diaminobenzidine (DAB) for 5 min. The reaction was stopped using distilled water.

The slides were counterstained with 7% Harris hematoxylin for 10 s, washed in running water, and dehydrated, diaphanized, and mounted with Enthellam^®^.

The same method was used to measure the percentage of immunostained cells. Histological sections were made using a semi-automatic microtome (Leica^®^) until the pulp chamber was visible in both specimens. Photomicrographs (U-TV0.63XC, Olympus^®^) were taken at 400x magnification (BX43, Olympus^®^ plus Olympus Soft Imagining LCMicro software) and mounted to access the entire area of the dental pulp of the right mandibular first molars. The images were then mounted and, using the Count Cells function, the number of odontoblasts and non-odontoblasts dental pulp cells was counted to measure the percentage of immunostained cells. We consider immunostained cells to be those with brown cytoplasm.^23^

### Statistical analysis

Data were presented as mean and standard error and analyzed using the Kruskal-Wallis test followed by Dunn’s post-hoc test. All analyses were performed using GraphPad Prism 5.0. statistical software, considering a 95% confidence level (p<0.05).

## Results

### Treatment with the Th17 response inhibitor digoxin reverses vascular changes in the dental pulp of rats treated with ZOL

**Figure 1:**
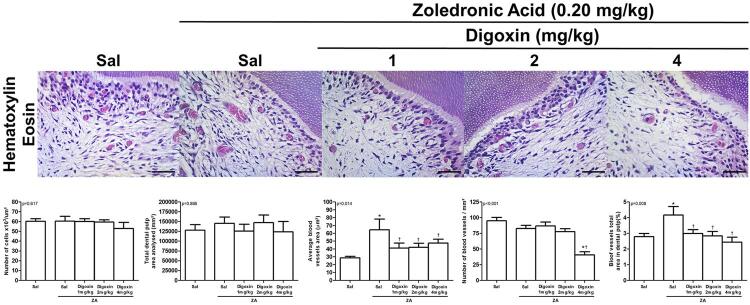
Cellular profile and vascular events in the dental pulp of rats chronically treated with ZOL and different doses of digoxin.Sal = saline solution

There were no significant differences in the pulp area of the groups treated with saline solution (127.974±14.131 µm^2^), ZOL (145.551±16.016 µm^2^), or co-treated with digoxin (p=0.866) 1 mg/kg (125.899±17.414 µm^2^), 2 mg/kg (147.451±19.104 µm^2^), or 4 mg/kg (124.033±26.424 µm^2^). Similarly, the cellularity of the dental pulp did not change significantly in the groups treated with saline solution (60±3 nuclei/µm^2^), ZOL (60±5 nuclei/µm^2^), or co-treated with digoxin (p=0.617) 1 mg/kg (60±2 nuclei/µm^2^), 2 mg/kg (60±2 nuclei/µm^2^), or 4 mg/kg (53±6 nuclei/µm^2^) (Table 1, Figure 1).

**Table 1:** Immunostaining score for Th17 response cytokines and associated transcription factors in odontoblasts and non-odontoblasts dental pulp cells from rats chronically treated with ZOL

	ZOL 0.20 mg/kg					
			Digoxin (mg/kg)			
	Sal	Sal	1	2	4	p-value
**Odontoblasts**						
IL-17	22.50±4.94	56.00±9.33*	66.67±8.38*	51.00±8.88*	30.82±6.33^†^	0.002
TNF-α	44.40±5.88	81.11±6.76*	64.62±7.89*	65.45±7.43*	38.33±6.26^†^	0.002
IL-6	18.33±1.12	20.00±6.45	46.15±6.46*^†^	60.00±9.63*^†^	54.17±5.57*^†^	<0.001
TGF-β	86.67±5.51	75.56±8.35	87.00±7.75	86.00±7.18	46.92±6.64*^†^	0.002
**Non-odontoblasts**						
IL-17	6.67±2.84	16.00±2.67*	18.33±1.67*	17.78±2.22*	15.56±2.94*	0.011
TNF-α	13.50±1.83	31.11±5.63*	32.08±4.14*	30.0±4.86*	22.92±5.45*	0.017
IL-6	10.00±3.01	6.67±3.33	15.38±2.43	16.00±2.67	15.00±2.61	0.140
TGF-β	27.50±3.91	23.64±4.32	21.00±3.79	23.00±3.00	24.62±3.12	0.850

*p<0.05 versus saline group; ^†^p<0.05 versus ZOL group; Kruskal-wallis/Dunn test (data expressed as mean±SE).

*p<0.05 versus NCG; †p<0.05 versus PCG; Kruskal-Wallis/Dunn test

Hematoxylin-eosin, 400x; Horizontal black bar = 50 µm; *p<0.05 versus NCG; †p<0.05, versus PCG (Kruskal-Wallis/Dunn).

The percentage of the pulp area filled with blood vessels of the rats treated with ZOL (4.16±0.54%) was significantly higher than that of NCG (2.79±0.20%). Rats treated with digoxin 1 mg/kg (2.99±0.24%), 2 mg/kg (2.84±0.28%), or 4 mg/kg (2.44±0.31%) showed an equal percentage reduction in the pulp area filled with blood vessels compared with the group treated with ZOL alone (p=0.008) (Table 1, Figure 1).

There was no significant difference between the number of blood vessels/µm^2^ in the groups treated with saline solution (95±5) and ZOL (83±5) and co-treated with ZOL and digoxin 1 mg/kg (87±6) or 2 mg/kg (78±4). However, rats co-treated with ZOL and the highest dose of digoxin (40±5) showed a significant reduction in the number of vessels/µm^2^ compared with the other groups (p<0.001) (Table 1, Figure 1).

The mean blood vessel area showed a significant increase compared with NGC (28.72±1.87 µm^2^) in the ZOL-treated group (64.56±12.56 µm^2^), with a significant reduction in DG1 (41.09±6.35 µm^2^), DG2 (42.18±5.12 µm^2^), and DG4 (47.61±4.94 µm^2^) (p=0.014) (Table 1, Figure 1).

### Digoxin treatment reduces IL-17-related TNF-α expression in the dental pulp of rats treated with ZOL

**Figure 2:**
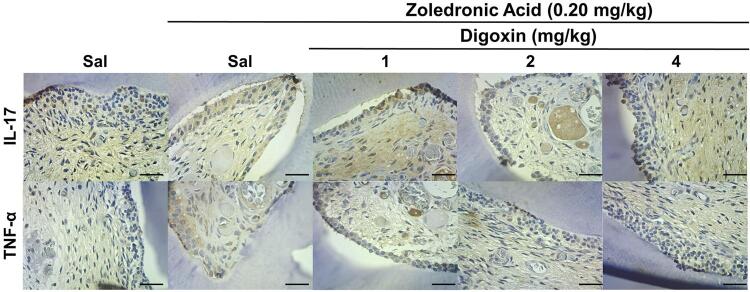
Immunostaining profile for IL-17 and TNF-α in the dental pulp of rats subjected to chronic ZOL treatment.

ZOL treatment significantly increased IL-17 expression in PCG compared with NCG. Rats co-treated with ZOL and digoxin 4 mg/kg showed significantly reduced immunoexpression scores for IL-17 compared with the ZOL-treated group (p=0.002) (Table 1, Figure 2).

Sal = saline solution

DAB-hematoxylin, 400x; Horizontal black bar = 50 µm. Black arrows = immunostained odontoblasts; White arrows = immunostained non-odontoblasts dental pulp cells.

Similarly, rats treated with ZOL showed higher immunoexpression scores for TNF-α than the control group treated with saline solution. The group treated with the highest dose of digoxin showed significantly reduced immunoexpression scores for TNF-α compared with rats treated with ZOL (p=0.002) (Table 1, Figure 2).

**Figure 3:**
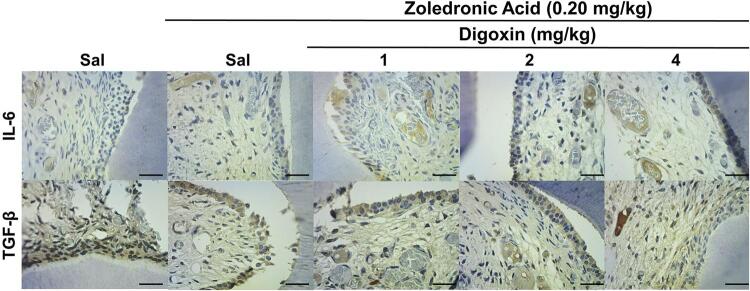
Immunostaining profile for IL-6 and TGF-β in the dental pulp of rats subjected to chronic ZOL treatment

Regarding IL-6, ZOL treatment did not significantly increase immunoexpression of IL-6 compared with NCG, but the groups co-treated with digoxin, regardless of dose, showed increased immunoexpression of IL-6 compared with the group treated with ZOL alone (p<0.001) (Table 1, Figure 3).

Sal = saline solution

DAB-hematoxylin, 400x; Horizontal black bar = 50 µm. Black arrows = immunostained odontoblasts; White arrows = immunostained non-odontoblasts dental pulp cells.

Immunoexpression of TGF-β was high in all groups. There was no significant difference between the immunoexpression of the saline-treated control group and the ZOL-treated group, but DG4 showed significantly reduced immunoexpression scores for TGF-β compared with the negative and positive control groups (p=0.002) (Table 1, Figure 3).

### Digoxin treatment alters the immunoexpression of IL-6 and TGF-β in the dental pulp in a manner unrelated to the Th17 response

Immunoexpression of IL-17 (p=0.011) and TNF-α (p=0.017) in non-odontoblasts pulp cells was significantly higher in the ZOL-treated group than in the negative control group, with no significant reduction after digoxin treatment (Table 1, Figure 2).

There was no significant difference in the expression of IL-6 (p=0.140) or TGF-β (p=0.850) in non-odontoblasts pulp cells (Table 1, Figure 2 and 3).

## Discussion

Inflammatory changes in the dental pulp of rats treated with ZOL have been previously described. Although not culminating in a state of inflammatory pulpitis, Silva, et al.^16^ (2016) and Silva, et al.^17^ (2019) reported increased expression of TNF-α, IL-1β, iNOS, COX-2, and toll-like receptors in odontoblasts after prolonged exposure to clinical doses of ZOL. Since there is no significant influx of inflammatory cells into the dental pulp, it seems that odontoblast metabolism is mainly involved in this process.

In this study, histological signs of vasodilation were observed with an increase in blood vessel area without changing the cellularity of the dental pulp. *In vitro* studies have shown that incubation with ZOL leads to cell death of odontoblasts,^15^ and *in vivo* studies have shown increased caspase-3 expression.^17^ However, the fact that there was no difference in the number of cell nuclei between the groups led to the hypothesis that pulpal inflammation associated with ZOL is not associated with significant cell death. This analysis also showed that there was no significant influx of inflammatory cells. As there is increased expression of inflammatory cytokines in the dental pulp,^16^ it seems that pulpal inflammation associated with ZOL is linked to the metabolism of the dental pulp cells themselves.

Odontoblasts are the cells responsible for dentinogenesis throughout life and promote the continuous metabolism of calcium and phosphate.^24^ Although dentin metabolism turnover is considerably slower than that of bone tissue, systemic changes that interfere with bone mineral density also interfere with dentin formation.^25^ Since ZOL has a high affinity for calcium and phosphate and also interferes with bone metabolism,^26^ it is likely that bisphosphonates directly interfere with odontoblasts, leading to this inflammatory process.

In rats treated with ZOL, there was a significant increase in TNF-α expression, especially in odontoblasts, as previously described.^16^ TNF-α is a potent vasodilator in the dental pulp, but the primary inflammatory stimuli that lead to the expression of these cytokines in the pulp tissue are transient.^27^ As ZOL is pro-inflammatory and spends long periods adhered to mineralized tissues,^26^ the chronification of the inflammatory process may alter the cytokine profile in the dental pulp, prolonging the half-life of TNF-α or its production.

No increase in IL-6 or TGF-β expression was observed in ZOL-treated odontoblasts, which is a necessary precondition for the activation of the Th17 response in lymphocytes.^28^ ZOL-dependent pulpal inflammation was not associated with the presence of inflammatory cells. Chronic exposure to ZOL is directly associated with IL-17 synthesis^29^ via activation of STAT1/IL-6.^30^ However, in the dental pulp, this process seems to use undescribed pathways, since IL-17 expression in the pulp is independent of standard inflammation cascades.^31^

IL-17 synthesis can change the natural course of several diseases, as the perpetuation of pro-inflammatory cytokine production is its most reported effect. By interacting with its receptors (IL-17R), IL-17 can activate TNFR synthesis. TNFR is TNF-α receptor and, by stabilizing the mRNA of this cytokine, it prolongs its action and increases the synthesis of this mediator. We observed that blocking IL-17 synthesis using digoxin also reduced TNF-α expression and vasodilation, but paradoxically increased IL-6 expression.^32^

Digoxin, besides being anti-inflammatory, has anti-vascular properties. Cardiac glycosides, including digoxin, ouabain, and proscillaridin A, inhibit hypoxia-inducible factor 1 (HIF-1α) protein synthesis and expression,^33^ which leads to vascular endothelial growth factor (VEGF) downregulation.^34^ The inhibition of other growth factors contributes to the antiangiogenic properties of digoxin, such as fibroblast growth factor (FGF) and epidermoid growth factor (EGF), both *in vivo* and *ex vivo*,^35^ which explains the reduction in TGF-β immunostaining showed in high doses of digoxin treatment and when added to the antiangiogenic action of ZOL, leading to considerable damage to the dental pulp.

The role of IL-17-ZOL-dependent pulp inflammation seems to be crucial, since inhibition by digoxin attenuated vasodilation, the microscopic change that indicates inflammation. Digoxin is a glycoside used to treat congestive heart failure and strongly inhibits RORγT, the main IL-17 transcription factor.^36^ The inhibitory action of digoxin on the Th17 response significantly improves autoimmune arthritis,^22^ and chronic use of this drug significantly reduces the risk of bone fractures due to osteoporosis.^37^ In this study, IL-17 is strongly associated with impaired mineralized tissue health^22,37^ and the maintenance of chronic inflammation in the dental pulp.

The main limitation of this study is the lack of evaluation of the Th1 response in the dental pulp. Although digoxin preferentially inhibits the Th17 response compared with the Th1 response, this aminoglycoside reduces the overproduction of some cytokines related to both immune responses, such as IL-1β, IL-6, IL-17, and IL-23.^38^ There is an overlap between the Th1 and Th17 immune responses during the development of immunity. However, toll-like receptors 2 and 4, which are overstained in odontoblasts after ZOL treatment,^17^ play an important role that favors the Th17 response, since while the Th1 response related to toll-like receptors is more important for intracellular microorganisms, the Th17 response related to toll-like receptors is more important for extracellular microorganisms.^39^ The main focus of these toll-like receptors is on odonoblasts.^40^

The reduction in IL-17 in digoxin-treated odontoblasts was not followed by reduced IL-17 immunoexpression in non-odontoblasts pulp cells. Thus, ZOL exerts a direct effect on odontoblasts via pathways independent of the classical activation by T helper lymphocytes, prolonging activation of IL-6 and TGF-β. However, it is still impossible to assess whether ZOL is incorporated into dentin as part of this change.

## Conclusion

Thus, we conclude that chronic ZOL administration induces IL-17-dependent vasodilation and pulp ectasia, and treatment with anti-RORyT digoxin partially reverted these outcomes. The anti-angiogenic effects of digoxin and the activity of odontoblasts seem to be responsible for this process. Since IL-17 expression in odontoblasts occurs independently of the classic IL-6 and TGF-β expression and the Th1 response overlaps with the Th17 response, further studies are needed to evaluate the pathways of activation of Th cells and IL-17 in odontoblasts and other dental pulp cells.
